# Supplementary material for: The relationship between screen time and gross motor movement: A cross-sectional study of pre-school aged left-behind children in China
Source: PLoS One. 2024 Apr 5;19(4):e0296862. doi: 10.1371/journal.pone.0296862 (PMC10997071; doi:10.1371/journal.pone.0296862)
Supplement: Attachment — Submitted filename: Response to Reviewers1.docx [file pone.0296862.s001.docx]

Dear reviewer:

Thank you very much for your meticulous review. Here are my answers to your questions and the relevant revisions to the article. The modifications have been highlighted in blue.

(i) The text needs to have an English language and textual review. In the introduction there is a lot of repetition of phrases with the same meaning. It can be more summarized.

The full text has been reviewed and revised.

(ii) The theme is very interesting, bringing the profile of an unusual and "official" population of child care, the so-called "left-behind children".

Left-behind children are commonly found in China or Southeast Asian countries and are a special group of people worthy of attention, so we have chosen them as research objects.

(iii) It was not clear in the consent form, how the parents were contacted to sign the participation of their children, under 36 years old, to participate in the application of the instrument. The amount is very high and this form of contact needs to be made clear.

We contact their guardians through the kindergarten.

(iv) I was surprised that a study that involved so many families and children did not have financial support for its realization.

It has now been added.

(v) The study is cross-sectional and, as mentioned, it cannot analyze the causality between whether the use of the screen is what causes the consequence in the gross motor pattern, in the child's development. However, I noticed that there was no separation by age, having grouped all the children in the analysis.

Age distribution has been added in Participants section.

(vi) Was a separate analysis made between the different ages, the time in which parents are away from their children?

This study did not analyze the departure time of parents of left-behind children. In follow-up research, we will further analyze variables such as the time when parents leave for children of different ages, whether both parents leave or one parent leaves.

(vii) How did the authors assess the quality of the information recorded by parents?

Through the kindergarten parents' meeting, the purpose of the study and the process of completing the questionnaire were explained in detail to all parents. After obtaining parents' informed consent, an online parent chat group was set up to guide children's parents in completing the questionnaire. The researcher checked the questionnaires' completion daily, reminded parents of the importance and authenticity of the questionnaires, and answered their questions.

(viii) Did the authors ask the question about the type of use of the cell phone (who owns it and if the parents control the time of use and observe the type of program watched)? In this case, as they are very young children, it is the parents who give them access to the screen.

The daily screen time information was collected by "the average number of minutes of screen time per day in the past 1d, except for class time", including the total time spent on TV, cell phone, and desktop computer. It is not just the amount of time children spend looking at cell phones but also the amount of time they spend watching TV.

Because smartphones have become popular in China, we did not investigate the type of phone. Guardians control the amount of time they spend on their cell phones to varying degrees (watching cartoons, mini-games, anime, etc.). But it's up to the guardians themselves. Many guardians use electronic products to keep their restless children quiet and avoid disturbing their time. Some parents even think that video games and videos can also promote the development of their children's intelligence and that playing them has no harm. However, they are oblivious to the dangers of their children's indulgence in electronic screens.

(ix) As it is a lower-income population, how many types of screens do children have access to at home? Was there variation between the time spent using the cell phone/tablet or TV? It is not clear from the text.

Smartphones and TVs are so cheap in China that they have long been popularized, and almost every family has TV and smartphone. Left-behind children are not all from low-income families. The phenomenon of parents leaving their children to go to work is common in China's towns and villages.

There is no difference between time spent using a cell phone/tablet or TV. We define screen time as including the total time spent on TV, cell phone, and desktop computer.

(x) In the discussion item, I considered that there was a limited search for updated references on the subject, which have been published in excellent journals, including pointing out differences between types of exhibitions

The references in the discussion section have been checked and updated.

(xi) Another strong limitation in the analysis was the lack of verification of screen time in the day care center by the evaluated children.

It has been added to the text. “Fifth, teachers teach according to the school's teaching plan, but due to the limited researchers, there are some difficulty in checking the daily screen usage with each teacher. Another strong limitation in the analysis was the lack of verification of screen time in the day care center by the evaluated children, and subsequent studies will improve this limit point.”

(xii) It was not clear in the text the details of how the instrument was applied and how the parents were interviewed by the four field researchers. Detailing this whole process is important.

It has been added to the text. Through the kindergarten parents' meeting, the purpose of the study and the process of completing the questionnaire were explained in detail to all parents. After obtaining parents' informed consent, an online parent chat group was set up to guide children's parents in completing the questionnaire. The researcher checked the questionnaires' completion daily, reminded parents of the importance and authenticity of the questionnaires, and answered their questions.

(xiii) In the conclusions, the authors should mention what kind of proposal they indicate for the participation of parents, in an intervention that seeks to guide them to reduce screen time and encourages more verbal contact with the environment, to reduce damage to gross motor coordination.

It has been added to the text. “This study can be used to inform teachers and families of the association between levels of screen time and the gross motor movement ability of children aged 3-6 years old. We suggest that guardian should guide and supervise children to reduce the screen time and encourage more verbal contact with the environment, to reduce damage to gross motor coordination.”

(xiv) The text could be more concise, but clearly showing the steps of the research, which can be reproduced by other researchers in the countries mentioned that it contains many "children left behind".

The investigation steps have been supplemented.

(xv) It was not clear in the study design how the sample that would be representative of this group of children was selected.

Henan is a large province of left-behind children; as of 2021, 1,595,600 rural left-behind children in compulsory education are in school, accounting for 10.70% of the total number of students in compulsory education, excluding preschool left-behind children (More than 120,000) . Henan Province has large -scale left -behind children, so we choose a city in Henan Province for investigation and research to ensure the representative of the sample.
